# Increasing availability of lower energy meals vs. energy labelling in virtual full-service restaurants: two randomized controlled trials in participants of higher and lower socioeconomic position

**DOI:** 10.1186/s12889-021-11007-0

**Published:** 2021-05-25

**Authors:** Lucile Marty, Sasha M. Reed, Andrew J. Jones, Eric Robinson

**Affiliations:** 1 Department of Psychological Sciences, University of Liverpool, Bedford Street South, Liverpool, L69 7ZA UK; 2 Centre des Sciences Du Goût et de l’Alimentation, Agrosup Dijon, CNRS, INRAE, Université Bourgogne Franche-Comté, 17 Rue Sully, 21065 Dijon Cedex, France

**Keywords:** Food choice, Energy labelling, Availability, Socioeconomic position, Restaurant

## Abstract

**Background:**

There are a range of interventions designed to promote healthier food choices in full-service restaurants. However, it is unclear how these interventions affect dietary choices in people of lower and higher socioeconomic position (SEP).

**Methods:**

A total of 2091 US participants recruited online completed Study 1 (*n* = 1001) and Study 2 (*n* = 1090). Recruitment was stratified by participant highest education level, resulting in higher SEP and lower SEP groups. In a between-subjects design, participants made hypothetical food choices (main dish, plus optional sides and desserts) from six restaurants menus in the absence vs. presence of menu energy labelling and from menus with baseline (10%) vs. increased availability (50%) of lower energy main dishes. Data were collected and analysed in 2019. Two studies were conducted in order to examine replicability and generalisability of findings across different restaurant menu types.

**Results:**

Across both studies, increasing the availability of lower energy main menu options decreased the average energy content of the ordered main dish (− 129 kcal, 95% CI [− 139; − 119]) and total energy ordered (− 117 kcal, 95% CI [− 138; − 95]) in both higher and lower SEP participants. Energy labelling significantly reduced the energy content of ordered main dishes in higher SEP participants (− 41 kcal, 95% CI [− 54; − 29]), but not lower SEP participants (− 5 kcal, 95% CI [− 22; 11]). However, energy labelling reduced total energy ordered (− 83 kcal, 95% CI [− 105; − 60]) irrespective of SEP.

**Conclusions:**

In two virtual experiments, increasing the availability of lower energy restaurant main menu options impacted on main menu dish choice and decreased total energy ordered irrespective of SEP. Energy labelling had a less pronounced effect on total energy ordered and had a larger impact on the energy content of main menu dish choice in higher as opposed to lower SEP participants.

**Trial registration:**

Clinicaltrials.gov NCT04336540 retrospectively registered (7 April, 2020).

**Supplementary Information:**

The online version contains supplementary material available at 10.1186/s12889-021-11007-0.

## Background

The eating out of home sector is now recognised as a potential area for public health intervention to improve diet and reduce obesity, in part because frequently eating outside of the home is associated with increased energy intake and obesity [1, 2]. Although the low nutritional quality of menu options in fast-food restaurants is well recognised [3], the full-service restaurant sector also warrants attention. A large number of dishes in full-service restaurants have been shown to have a high energy content and a number of studies indicate that full-service restaurants tend to provide meals that are significantly higher in energy content than fast-food restaurants [4–7].

There is a range of interventions that can be implemented in full-service restaurant settings to improve the nutritional quality of food consumed [8, 9]. Menu energy labelling has been mandated recently in US restaurants [10], regions of Canada [11] and Australia [12] and is currently being considered in the UK [13]. Evidence to date is mixed on the effectiveness of energy labelling, with both virtual and real-world studies finding evidence of either a small effect or no effect on energy ordered. Recent meta-analyses have reported that energy labelling was associated with a − 7.63 kcal difference (95% CI [21.02, 5.76]) or a 0.03 difference in energy ordered (95% CI [− 0.96, 0.89]) in restaurant settings [14, 15]. In line with the latter finding, in a recent virtual fast-food restaurant experiment there was no evidence that energy labelling decreased energy ordered [16].

There is also a small amount of emerging evidence that the effectiveness of energy labelling on energy ordered may primarily be observed among people of higher socioeconomic position (SEP) and not lower SEP [17]. This may be the case because energy labelling is an example of an information-based intervention, as one of its primary (but not only) purposes is to provide consumers with information that they can then use to change their behavior [18]. Because people of lower SEP report being less motivated by health and weight control when making food choices [19, 20] and may be more likely to engage in impulsive behaviors [21] than higher SEP populations (e.g. acting based on other food motives or impulse rather than health or weight concerns), energy labelling may be less effective in lower as opposed to higher SEP populations.

An alternative to energy labelling is to directly change ‘structural’ elements of food environments. One example of this type of approach is increasing the availability (i.e. proportion) of menu options that are lower in energy content. The best available evidence to date from real-world and virtual studies suggests that increasing the availability of lower energy options decreases energy ordered, but there is very little research that has tested this intervention approach in isolation in full-service restaurant settings [22, 23]. It has been hypothesised that because approaches like this do not rely on individual agency, unlike information-based approaches, they may be more equitable for people of higher and lower SEP [24, 25]. In a recent virtual fast-food restaurant experiment, we found convincing evidence that increasing the availability of lower energy options decreased energy ordered to a similar degree in participants of lower vs. higher SEP [26]. However, there have been few other studies that have been designed to address this question (e.g. recruiting sufficient numbers of both lower and higher SEP participants) and none that we are aware of in full-service restaurant settings.

There is limited evidence on the likely effects that different interventions in full-service restaurants have on food choice and whether interventions may inadvertently widen inequality by benefiting people of higher, rather than lower SEP. In the current research, we compared the effect that energy labelling and increasing the availability of lower energy main menu options have on the food choices and energy ordered of higher and lower SEP participants in two online virtual experiments. Because education level is a known social determinant of food choice motives [19, 20], diet and obesity [27–29], consistent with other research on nutrition [30] we based recruitment of stratification of higher vs. lower SEP on participant education level. US participants made hypothetical menu orders from different restaurant menus and we conducted two studies to examine generalisability (some restaurant cuisines differed between the two studies) and replicability of findings. Recent findings have suggested that rather than only altering main meal food choices, energy labelling may in part reduce energy ordered by decreasing the amount of additional food customers order [31]. To represent a typical full-service restaurant setting, participants chose a main meal dish and had the option of ordering additional sides and desserts, as this allowed us to examine the effect of interventions on food choice (amount of energy in main dish) and total energy ordered. Using a similar design, we recently examined food choices in a virtual fast food restaurant environment [16]. However, relatively few experiments have examined energy labelling in the context of full-service restaurants [32] and there is some evidence that energy labelling may be less effective in fast-food restaurants than other outlets, such as full-service restaurants [33]. Therefore, the present work builds on our earlier work by examining food choice in a different environment (full-service restaurant environment with multiple cuisine menu types) where there was less constraints on the choice (i.e. in the present study participants could include optional sides and desserts) than in the study we previously conducted at a virtual fast-food restaurant (participants had to choose a main, side and drink) [16]. We also included psychological measures (e.g. food choice motives, impulsivity), in order to explore reasons why energy labelling may be less effective in lower than higher SEP participants.

## Methods

### Study samples

We conducted two randomized controlled experiments. Participants were recruited for monetary compensation through the online platforms Prolific Academic [34] or Turk Prime [35] between August and November 2019. Data were analysed in December 2019. Eligibility criteria were: US residents, 18 years or above, fluent in English, access to a computer with an internet connection, no dietary restrictions. If a participant failed one or more attention check they were screened out and their data was excluded (see Additional file 1 – section 1). We stratified recruitment by gender (approx. 50/50) and highest educational qualification (approx. 40% high school or less, 60% above high school) so that our sample was broadly representative of US adults [36]. The Health and Life Sciences Research Ethics Committee at the University of Liverpool approved the research (reference: 4612) and consent (informed) was required from all the participants before beginning the study. Participants were made aware prior to consent that the study was about food choices at restaurants but were not informed of the study aims or hypotheses.

### Design overview

Participants were assigned randomly to one of four conditions using a 2 × 2 between-subjects design: ‘baseline availability’ and ‘no energy labelling’ (A- L-), ‘baseline availability’ and ‘energy labelling’ (A- L+), ‘increased availability of lower energy options’ and ‘no energy labelling’ (A+ L-), ‘increased availability of lower energy options’ and ‘energy labelling’ (A+ L+). We used a randomisation allocation (administered in the Qualtrics survey platform) of 1:1:1:1.

### Measures and procedure

In a hypothetical meal choice task, participants were asked to select meals from six different menus. In Study 1, menus of cuisines participants were familiar with (American, Mexican and Italian) and less familiar with (Lebanese, Peruvian and Moroccan) were chosen. Unfamiliar cuisines were replaced by more common cuisines in Study 2, as we found no evidence that results were dependent on cuisine familiarity in Study 1: Chinese, Japanese and Greek (see Additional file 1 – section 2 for a detailed description of cuisine familiarity). The design of the restaurants menus was based on online menus of popular US restaurants. Each menu consisted of ten main dishes, in addition to five sides and five desserts. Menu option names, description, prices and energy content (for L+ conditions) were taken from actual restaurant menus. The order of the dishes presented in each main menu was counterbalanced (see Additional file 1 – section 3).

We categorised main dishes as ‘lower energy’ (LE) ≤ 600 kcal vs. ‘higher energy’ (HE) > 600 kcal based on dietary recommendations for US adults (i.e., on average 2000 kcal per day) assuming a daily diet of three main meal occasions, each accounting for 20–35% of daily energy intake, and one or two snacking occasions, each accounting for 5–10% of daily energy intake [37]. Consistent with actual menus [4] in the baseline availability conditions (A-), 1/10 main dishes on the menus were LE and 9/10 were HE. In the increased availability of lower energy options conditions (A+), the proportion of LE main dishes was increased to 5/10 by replacing four of the HE main dishes with LE main dishes, whilst holding the price of replaced menu items the same. The menu items with the highest and the lowest energy content remained the same in all conditions and the difference in average energy of the mains between A- and A+ conditions was similar across the six menus (approx. 200 kcal). In the energy labelling conditions (L+), consistent with US recommendations energy in kcal was next to each menu option and reference information on energy requirements was clearly displayed at the bottom of the menus. In the ‘no energy labelling’ conditions (L-), no kcal information and no reference information on energy requirements was included. The sides and desserts options remained the same across all conditions and for consistency energy in kcal was included in the energy labelling conditions (L+). An example menu (American cuisine, A- L+ condition) is provided in Additional file 1 – section 4.

After providing informed consent, participants completed demographic-based questions. See Additional file 1 – section 5 for items. In the next part of the study participants made hypothetical meal choices from each of the six restaurant menus. They were first shown an image of the restaurant from the outside, before being shown an image of the inside of the restaurant and then the menus. Participants chose one main meal and were asked if they would like to order any extra sides or desserts; they could order zero or up to five sides and desserts. Next, participants completed questionnaires about their food choices motives: single-item Food Choice Questionnaire [38] (Study 1), health motivation (6 items – Cronbach’s α = 0.90) and weight motivation (4 items – Cronbach’s α = 0.82) subscales from the Food Choice Questionnaire [39] (Study 2); about nutrition knowledge (Study 2): general nutrition knowledge questionnaire (20 items – Cronbach’s α = 0.80) [40]; about impulsivity (Study 2): Barratt Impulsiveness Scale (30 items – Cronbach’s α = 0.83) [41] and a 5-trial delay discounting task [42] where participants received five questions on whether they prefer a smaller amount of money ($5) now or a larger amount ($10) at variable delays depending on previous choice (a behavioral measure of impulsivity). For this task, the discount rate (k) was calculated as the inverse of the Effective Delay 50% (ED_50_) which is the delay (in days) that discounts the value of the delayed reward by 50% [43]. Finally, participants were asked to describe what they thought the study aims were. If a participant reported either the impact on food choice of energy labelling or mentioned availability of lower energy options in their answer they were classed as being aware of study aims. This was coded by two researchers, with any discrepancies solved by discussion with another independent researcher. Participants next completed five questionnaire items about the restaurant menus. See Additional file 1 – section 6 for questionnaire items. Finally, participants were debriefed and compensated for their time.

### Statistical analyses

Pre-registered analysis protocols are available online (https://osf.io/amdnq/). The measure of SEP used in our primary analyses was the highest educational qualification (categorical variable: lower ≤ high school vs. higher > high school). Primary analyses for Study 1, Study 2 and pooled data (combining Study 1 and Study 2 data, controlling for study) were linear mixed models testing the effect of energy labelling (absent vs. present), availability (baseline vs. increased availability of lower energy options), highest educational qualification (lower vs. higher) and labelling*highest educational qualification and availability*highest educational qualification interactions on energy of the main for the six menus, with random effects of participant and menu to account for correlation between repeated ordering by the same participant and across menus. Stratified models on highest educational qualification were run to examine interactions with education level. If a participant did not complete the study in full then their data was not included in any analyses. Sensitivity analyses conducted involved repeating the above analyses: 1/ after excluding aim guessers, 2/ treating education level as a continuous measure (see Additional file 1 – section 7). We also used the same analysis approach to examine total energy ordered (mains, sides and desserts) on the pooled data. Additionally, if we found evidence that the effect of an intervention on energy ordered was moderated by SEP, moderated mediation was tested by estimating the difference of the conditional indirect effect of highest educational qualification on the energy of the main through 1/ health motivation (Study 1 and Study 2), 2/ weight control motivation (Study 1 and Study 2), 3/ general nutrition knowledge (Study 2), 4/ trait impulsivity (Study 2) and 5/ discount rate (Study 2) between labelling and no labelling conditions. We used the PROCESS macro (Model 15) on SAS version 9.3 that provides asymmetric bias-corrected bootstrap confidence intervals for inference about the conditional indirect effects using 5000 bootstrap samples [44]. SAS version 9.3 (SAS Institute, Inc., 2012 SAS® 9.3. Cary, NC) was used for the majority of analyses (described above). Significance levels of *p* <  0.05 were used for the main and sensitivity analyses, and for the purpose of secondary analyses *p* <  0.01 was used to adjust for multiple tests. To further examine evidence for the hypotheses, Bayesian analyses using default priors were performed on pooled data across studies (JASP Version 0.9.2).

A 7% reduction in energy purchased at restaurants attributable to energy labelling was reported in a recent systematic review and meta-analysis [32]. Studies that have examined the impact that altering availability of lower-energy options have tended to report similar sized or larger sized effects than that of energy labelling [45, 46]. Therefore, we powered each study (α = 0.05, 1 – β = 0.8) to detect a 2% reduction in energy ordered as a result of energy labelling or availability and an additional 2% energy reduction due to an interaction with highest educational qualification (*n* = 1000 participants). See Additional file 1 – section 8 for full power calculation information.

## Results

**Fig. 1:**
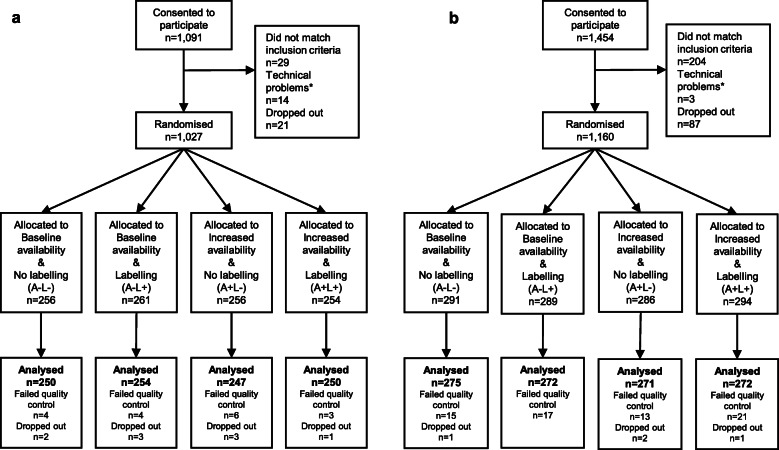
Study flow charts. **a**, Study 1. **b**, Study 2

Across both studies, 2251 participants consented to take part and data from 2091 completing participants was analysed (Fig. 1). A summary of participants’ characteristics is presented Table 1. The final samples for Study 1 and Study 2 were similar in demographic characteristics and across experimental conditions (Additional file 1 – section 9).

**Table 1:** Participants’ characteristics

	Study 1 (***n*** = 1001)	Study 2 (***n*** = 1090)
**Age, years, mean (SD)**	35.3 (12.7)	44.9 (18.5)
**Gender**^**a**^, female, n (%)	532 (53.2)	624 (57.2)
**Ethnicity, n (%)**		
*White, non-hispanic*	772 (77.1)	916 (84.0)
**BMI, kg/m2, mean (SD)**	28.2 (7.75)	28.1 (7.43)
*Missing, implausible*^b^*, n (%)*	79 (7.9)	20 (1.8)
**Employment status, n (%)**		
*Full or part-time*	606 (60.5)	482 (44.2)
*Student*	78 (7.8)	57 (5.2)
*Retired*	36 (3.6)	248 (22.7)
*Sick or disabled*	30 (3.0)	75 (6.9)
*Looking after home*	122 (12.2)	127 (11.7)
*Other unemployed*	129 (12.9)	101 (9.3)
**Highest educational qualification binary, n (%)**		
*Low (≤ High-school completion)*	351 (35.1)	388 (35.6)
*High (> High-school completion)*	650 (64.9)	702 (64.4)
**Years of higher education, mean (SD)**	5.88 (2.37)	5.9 (2.57)
**Household income, $, mean (SD)**	56,432 (45831)	53,165 (49129)
**Subjective socioeconomic status, mean (SD)**^c^	4.68 (1.75)	4.98 (1.88)
**Dieting status, yes, n (%)**	138 (13.8)	116 (10.6)

^a^Participants were asked about their gender, ^b^BMI implausible values: BMI <10 or BMI > 60 [47], ^c^Higher scores indicate higher perceived socioeconomic status, scale range 1–10

### Energy content of main meal dish ordered

**Fig. 2:**
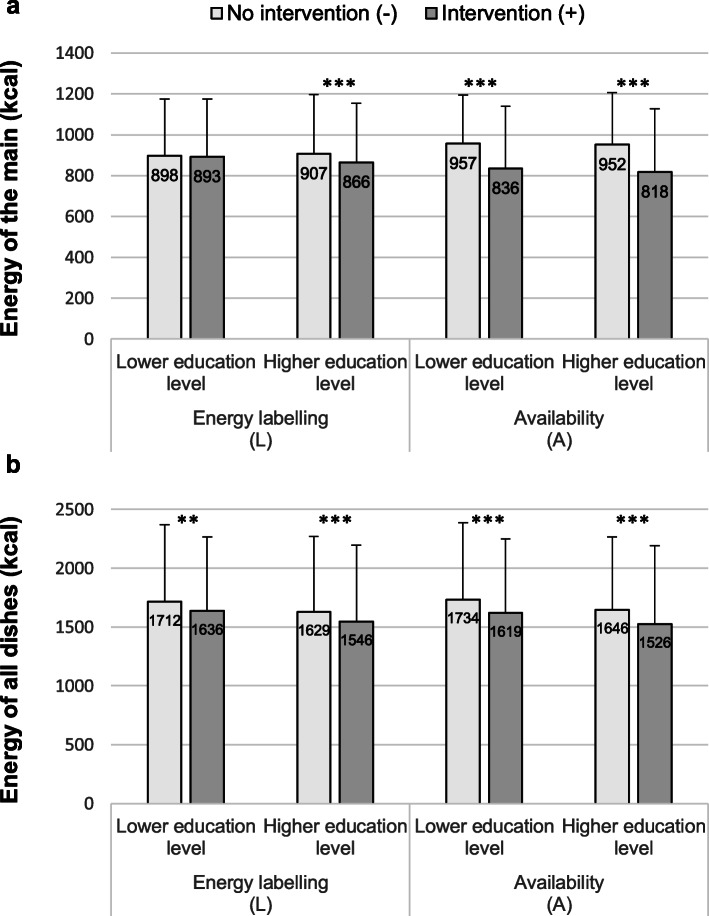
Mean energy (+ SD) from the main meal dish ordered (**a**) and from all dishes orders (**b**) across the two studies

Statistical models are reported in Table 2. In Study 1 there was a significant main effect of availability on energy of ordered main, but no interaction with highest educational level. There was also a significant main effect of energy labelling on energy of ordered main and this was qualified by an interaction with highest education level, whereby labelling only decreased energy of ordered main in participants of higher educational qualification. Cuisine familiarity did not influence the effect of the interventions (Additional file 1 – section 10). The same pattern of results was observed in Study 2 and it was unchanged in sensitivity analyses (Additional file 1 – section 11). Analyses on the pooled data confirmed the pattern of results found in Study 1 and Study 2 using both frequentist analyses (Table 2 and Fig. 2) and Bayesian analyses (Additional file 1 – section 12). Across the two studies, participants ordered on average a main meal dish of 954 kcal (95% CI [948; 960]) in A- conditions and 824 kcal (95% CI [817; 832]) in A+ conditions; the difference between A+ and A- was − 129 kcal (95% CI [− 139; − 119]). They ordered on average a main meal dish of 904 kcal (95% CI [897; 911]) in L- conditions and 875 (95% CI [868; 882]) in L+ conditions; the difference between L+ and L- was − 29 kcal (95% CI [− 39; − 19]).

**Table 2:** Fixed effect statistics of complete and stratified linear mixed models with participant and menu effect as random, dependant variable: energy of the main

	**Study 1**				
	**Type III tests**		***Estimate***^a^	***95% LCL***	***95% UCL***
**Model**	***F***	***p***			
**Complete (*n*** **= 1001)**					
(Intercept)			962.20	850.71	1073.68
Availability	256.43	< 0.001	− 128.41	− 153.81	− 103.01
Labelling	9.25	0.002	−3.53	−28.93	21.87
Education	1.62	0.203	11.00	−16.34	38.33
Availability*Education	< 0.01	0.968	−0.64	−32.16	30.88
Labelling*Education	6.78	0.009	−41.85	−73.37	−10.33
**Education = ‘low’ (*n*** **= 351)**					
(Intercept)			962.20	849.06	1075.33
Availability	105.86	< 0.001	−128.41	− 152.89	−103.93
Labelling	0.08	0.778	−3.53	−28.01	20.95
**Education = ‘high’ (*n*** **= 650)**					
(Intercept)			973.19	864.55	1081.84
Availability	176.88	< 0.001	−129.05	− 148.08	− 110.03
Labelling	21.87	< 0.001	−45.38	−64.40	−26.35
	**Study 2**				
	**Type III tests**		***Estimate***	***95% LCL***	***95% UCL***
**Model**	***F***	***p***			
**Complete (n = 1090)**					
(Intercept)			957.16	917.50	996.83
Availability	243.80	< 0.001	− 115.16	− 140.67	−89.66
Labelling	7.24	0.007	−6.33	−31.90	19.23
Education	2.08	0.149	15.20	−12.12	42.52
Availability*Education	1.98	0.160	−22.80	−54.58	8.98
Labelling*Education	3.65	0.056	−31.03	−62.87	0.81
**Education = ‘low’ (*n*** **= 388)**					
(Intercept)			957.16	923.58	990.74
Availability	82.46	< 0.001	−115.16	−140.04	−90.29
Labelling	0.25	0.619	−6.33	−31.26	18.60
**Education = ‘high’ (*n*** **= 702)**					
(Intercept)			972.36	933.28	1011.44
Availability	198.04	< 0.001	− 137.96	− 157.18	− 118.74
Labelling	14.51	< 0.001	−37.36	−56.59	−18.13
	**Pooled data**				
	**Type III tests**		***Estimate***	***95% LCL***	***95% UCL***
**Model**	***F***	***p***			
**Complete (*n*** **= 2091)**					
(Intercept)			967.55	901.39	1033.71
Availability	498.83	< 0.001	− 121.44	−139.44	− 103.43
Labelling	16.49	< 0.001	−5.11	−23.13	12.90
Education	3.72	0.054	13.16	−6.17	32.48
Availability*Education	1.14	0.285	−12.20	−34.59	10.19
Labelling*Education	10.01	0.002	−36.16	−58.56	−13.76
Study	7.61	0.006	−19.81	−33.89	−5.73
**Education = ‘low’ (*n*** **= 739)**					
(Intercept)			970.70	903.09	1038.31
Availability	185.94	< 0.001	−121.44	−138.90	−103.98
Labelling	0.32	0.570	−5.07	−22.56	12.41
Study	4.08	0.044	−23.63	−46.57	−0.69
**Education = ‘high’ (*n*** **= 1352)**					
(Intercept)			978.65	914.01	1043.29
Availability	374.97	< 0.001	− 133.64	− 147.16	− 120.11
Labelling	35.74	< 0.001	−41.27	−54.80	−27.73
Study	3.57	0.059	−17.06	−34.76	0.64

^a^Intercept estimate for the reference group: baseline availability, no labelling, low education level (complete models), study 1 (pooled data); estimates of the fixed effects must be interpreted as differences with the reference group

### Energy content of all dishes ordered

Analyses on pooled data showed a significant main effect of availability and labelling on energy of all dishes ordered. Across the two studies, participants ordered on average 1677 kcal in total (95% CI [1661; 1692]) in A- conditions and 1559 kcal (95% CI [1543; 1576]) in A+ conditions; the difference between A+ and A- was − 117 kcal (95% CI [− 138; − 95]). They ordered on average 1660 kcal (95% CI [1644; 1676]) in the L- conditions and 1577 kcal (95% CI [1561; 1593]) in the L+ conditions; the difference between L+ and L- was − 83 kcal (95% CI [− 105; − 60]). There was also a significant main effect of highest educational qualification, whereby participants of lower educational level ordering more energy than participants of higher educational level (Additional file 1 – section 13). No interaction was found between availability or labelling and highest educational level (Fig. 2b). The lack of interaction effect between labelling and highest educational level on energy of all dishes ordered appeared to be due to a significant labelling effect in both participants of lower and higher education when ordering sides and desserts (Additional file 1 – section 14).

### Moderated mediation

In both Study 1 and Study 2 the primary analysis suggested that highest educational qualification moderated the effect of labelling on energy of the main. Detailed moderated mediation analyses results are reported in Additional file 1 – section 15. In summary, in Study 1, no significant evidence for moderated mediation through health or weight control motivation was found when they were measured with a single item from the single Food choice motives questionnaire [38]. In Study 2, using the more detailed food choice motives questionnaire the indirect effect of educational qualification on energy of ordered mains through weight control motivation was significantly moderated by labelling. Therefore, energy labelling being associated with less energy ordered in participants of higher but not lower educational qualification was in part explained by lower levels of weight control motivation among less educated participants. See Additional file 1 – section 15 for detailed statistics. There was no evidence of moderated mediation through general nutrition knowledge, trait impulsivity or delay discounting.

## Discussion

In two virtual online experiments we examined the effect of providing menu energy labelling and increasing the availability of lower energy main menu dishes on main menu food choices and total amount of energy ordered by participants of lower and higher SEP. Results were consistent across the two studies. Pooled analysis of the two studies indicated that increasing the availability of lower energy main menu dishes (mains with ≤600 kcal) from 10 to 50% resulted in participants choosing a main menu dish with 130 fewer kcal on average, and reduced the total amount of energy ordered (main dish in addition to optional sides and dessert) by 118 kcal. These effects were not moderated by SEP. Providing energy labelling significantly affected energy content of chosen main menu dish in participants of higher SEP (− 41 kcal), but did not significantly affect the energy content of main menu dishes ordered by lower SEP participants (− 5 kcal). Energy labelling did result in a reduction to total energy ordered and this effect was not moderated by SEP.

In two recent similar experiments that examined ordering behavior in a fast-food restaurant environment, we did not find evidence that energy labelling reduced energy ordered [16]. In the present experiments we did find evidence of ordering being affected by energy labelling and these findings are in line with a recent systematic review which suggests that energy labelling may have a smaller impact on ordering behavior in fast-food restaurant settings, compared to other outlets, such as full-service restaurants [33]. These divergent findings may be in part explained by sit-down restaurants having more very high energy menu options than fast-food restaurants (meals ≥1000 kcal) and therefore more likely to be avoided in the presence of energy labelling [4]. However, there are other differences between fast-food and full-service restaurants and it would be informative to understand the conditions under which energy labelling is likely to impact on consumer behavior.

Although energy labelling did not affect initial food choice (e.g. choice of higher vs. lower energy mains) in lower SEP participants, labelling did affect the extent to which lower SEP participants ordered additional food items (as indicated by the effect of energy labelling on total energy ordered not being moderated by SEP). This finding is consistent with a recent real-world study examining fast food purchases in which a reduction to energy ordered was in part explained by customers ordering fewer menu items per visit [31]. These findings highlights the need for studies to examine both the types and amounts of food people order (e.g. number of side dishes) when testing the efficacy of energy labelling [31]. We found evidence in Study 2 that the tendency for energy labelling to affect the main meal choices of higher, but not lower SEP participants was in part explained by SEP differences in food choice motives. Higher SEP was associated with greater weight control motives (self-reported) when making everyday food choices and weight control motives predicted participants choosing a main with less energy in response to energy labelling. These findings are consistent with the proposition that information-based interventions may be more likely to benefit higher SEP than lower SEP populations because they require a higher level of engagement and agency (i.e. being motivated to change behavior). However, when examining total energy ordered, we found no statistical evidence of energy labelling benefiting higher SEP participants more than lower SEP. Unlike energy labelling, increasing the availability of lower energy menu options resulted in participants of both higher and lower SEP choosing a lower energy main and ordering less energy overall. This latter finding is consistent with the notion that ‘structural’ interventions are more likely to be equitable than information-based interventions [24, 25].

Strengths of the present research include the use of a large sample of lower and higher SEP participants, pre-registration of analyses and replication of findings across independent samples of participants. Further strengths of the study are that we were able to set tightly controlled experimental conditions (e.g., we held price and number of options constant across conditions and counterbalanced presentation of dishes) and test psychological mechanisms as explanations of SEP differences in response to energy labelling interventions. If replicated in real-world settings the findings of the present research may have implications for public health interventions. In particular, it will be important for evaluations of menu energy labelling policies to consider their impacts on people of lower and higher SEP. For example, in situations where people primarily choose between two similar dishes (e.g. choice of sandwich filling), energy labelling may primarily benefit people of higher SEP. The present research also identified that energy labelling is less impactful on main dish choice in lower SEP populations because food choice motives differ based on SEP. Based on this finding, any impact of energy labelling in lower SEP groups may be maximised by introducing paired initiatives that encourage people to be more motivated by weight control when making food choices. It is also important to note that energy labelling may improve diet without changing restaurant ordering behavior, such as labelling laws resulting in restaurants reformulating dishes to reduce energy or by decreasing energy consumed at home, both of which may or may not differ according to SEP. Yet, the relatively small effects (compared to altering availability) of energy labelling observed in the present studies indicate that policies in addition to energy labelling will be required to substantially reduce population level energy intake.

### Limitations

Although menus were based on popular US cuisines from common US restaurants, studies were online virtual experiments. In addition, because participants were not spending money and were not required to eat the food choices made it is unclear whether the same pattern of results would be observed in real-world conditions. Likewise, although we stratified recruitment to approximately resemble key features of the US population (e.g. SEP), the types of people who participate in online experiments may differ to the general population. We aimed to recruit a sample largely representative of the US adult population, but our sample had slightly more females (55% female, 45% male) and more highly educated participants than planned. Like other energy labelling research, the present studies also provide information about short-term choice behavior and it will be important to understand effects of energy labelling over longer periods of time and any effects on other behaviors (e.g. physical activity).

## Conclusions

In two virtual experiments, increasing the availability of lower energy restaurant main menu options impacted on main menu dish choice and decreased total energy ordered irrespective of SEP. Energy labelling had a less pronounced effect on total energy ordered and had a larger impact on the energy content of main menu dish choice in higher as opposed to lower SEP participants.

## Supplementary Information

**Additional file 1.** All additional materials and data.

## Data Availability

The datasets generated and analysed during the current study are available in the OSF repository: https://osf.io/amdnq/.

## Abbreviations

BMI: Body mass index
ED: Effective delay
HE: Higher energy
LE: Lower energy
SEP: Socioecomonic position
