# Supplementary material for: Increasing availability of lower energy meals vs. energy labelling in virtual full-service restaurants: two randomized controlled trials in participants of higher and lower socioeconomic position
Source: BMC Public Health. 2021 May 25;21:975. doi: 10.1186/s12889-021-11007-0 (PMC8147368; doi:10.1186/s12889-021-11007-0)
Supplement: Supplementary file 1 — Additional file 1. All additional materials and data. [file 12889_2021_11007_MOESM1_ESM.docx]

**Additional file 1 (Appendix)**

**Table of contents**

[**1.** **Quality control (attention check) questions** 2](#_Toc35966780)

[**2.** **Cuisine familiarity** 3](#_Toc35966781)

[**3.** **Counterbalancing order of dishes** 4](#_Toc35966782)

[**4.** **Example menu** 5](#_Toc35966783)

[**5.** **Demographic-based questionnaire** 7](#_Toc35966784)

[**6.** **Restaurants questionnaire** 8](#_Toc35966785)

[**7.** **Alternative continuous measure of education level** 11](#_Toc35966786)

[**8.** **Power calculation** 12](#_Toc35966787)

[**9.** **Participants characteristics overall and across experimental conditions** 12](#_Toc35966788)

[**10.** **Effect of the interventions and cuisine familiarity (Study 1)** 14](#_Toc35966789)

[**11.** **Sensitivity analyses** 16](#_Toc35966790)

[*11.1.* *Excluding aim guessers* 16](#_Toc35966791)

[*11.2.* *Substituting the categorical variable ‘highest educational qualification’ by the continuous composite variable ‘level of education’* 17](#_Toc35966792)

[**12.** **Bayesian analyses on pooled data** 19](#_Toc35966793)

[**13.** **Effect of the interventions on energy content of all dishes ordered (pooled data)** 20](#_Toc35966794)

[**14.** **Effect of the interventions on energy content of extra dishes ordered only – sides and desserts (pooled data)** 21](#_Toc35966795)

[**15.** **Moderated mediation analyses** 22](#_Toc35966796)

**Tables of figures**

[**Figure S1.** American main dishes menu in the A-L+ condition (version 1/6) 6](#_Toc36139097)

[**Figure S2.** Moderated mediation conceptual diagram 22](#_Toc36139098)

[**Figure S3.** Moderated mediation with three mediators conceptual diagram 24](#_Toc36139099)

**Table of tables**

[**Table S1.** Level of familiarity for each cuisine (n=1,001) 3](#_Toc36139104)

[**Table S2.** Classification of the America menu items (A- conditions) 4](#_Toc36139105)

[**Table S3.** Description of ‘top’, ‘middle’ and ‘bottom’ positions on the menus 4](#_Toc36139106)

[**Table S4.** Counterbalanced order of the dishes presented in each main menu 5](#_Toc36139107)

[**Table S5.** Demographic-based questionnaire 7](#_Toc36139108)

[**Table S6.** Descriptive analyses for each item of the debriefing questionnaire 8](#_Toc36139109)

[**Table S7.** ANOVA models, dependant variables: debriefing questionnaire items 10](#_Toc36139110)

[**Table S8.** Study 1 participants characteristics 12](#_Toc36139111)

[**Table S9.** Study 2 participants characteristics 13](#_Toc36139112)

[**Table S10.** Fixed effect statistics of complete and stratified linear mixed models with participant and menu effect as random, dependant variable: energy of the main. 15](#_Toc36139113)

[**Table S11.** Fixed effect statistics of complete and stratified linear mixed models without aim guessers with participant and menu effect as random, dependant variable: energy of the main. 16](#_Toc36139114)

[**Table S12.** Fixed effect statistics of complete and stratified linear mixed models with the composite score ‘level of education’ with participant and menu effect as random, dependant variable: energy of the main. 18](#_Toc36139115)

[**Table S13.** Bayes factors for comparison of the likelihood of energy of the main pooled data under several models 19](#_Toc36139116)

[**Table S14.** Fixed effect statistics of complete and stratified linear mixed models with participant and menu effect as random, dependant variable: total energy ordered (mains, sides and desserts) 20](#_Toc36139117)

[**Table S15.** Fixed effect statistics of complete and stratified linear mixed models with participant and menu effect as random, dependant variable: extra energy ordered (sides and desserts) *–* only orders with extra sides or extra desserts 21](#_Toc36139118)

[**Table S16.** Conditional direct and indirect effects of education on average energy of main menu choice 22](#_Toc36139119)

[**Table S17.** Pearson’s correlations between health motivation, weight control motivation and impulsivity scores adjusted for educational qualification (n=1,090) in Study 2 24](#_Toc36139120)

[**Table S18.** Conditional direct and indirect effects of education on average energy of main menu choice in a joint model including three mediators: health motivation, weight control motivation and impulsivity. 25](#_Toc36139121)

1. **Quality control (attention check) questions**

We included three quality control (attention check) questions in Study 1 and Study 2. The first quality control item was “This is an attention check. How many times have you visited the planet Mars?” and possible responses were ‘Several times’, ‘Just once’ and ‘Never’. The second item was “This is an attention check. Please click the fifth option on this menu” with ten response options. The third item in Study 1 was “This is an attention check. Please choose the answer 2 ‘Not important’” with possible responses on a 7-point scale from 1 ‘Not at all important’ to 7 ‘Very important’. The third item in Study 2 was “This is an attention check. Please choose the answer 2 ‘A little important’” with possible responses on a 4-point scale from 1 ‘Not at all important’ to 4 ‘Very important’. Participants were excluded if they gave any responses other than ‘Never’ to the first item, other than the fifth option to the second item, other than ‘Not important’ or ‘A little important’ to the third item.

1. **Cuisine familiarity**

For Study 1 the choice of the cuisines was based on a recent study where 1,184 US adults were asked which of 34 national cuisines they had tried and whether they liked or disliked them^1^. For Study 1 we selected three familiar (tried by at least 90% of US people: American, Mexican and Italian cuisines) and three unfamiliar cuisines (never tried by approximately 50% of US people: Lebanese, Peruvian, Moroccan cuisines). For Study 2, the three unfamiliar cuisines were replaced by more familiar cuisines (tried by at least 75% of US people: Chinese, Japanese and Greek).

A summary of the answers to the questions “Have you ever tried [American, Mexican, Italian, Lebanese, Peruvian, Moroccan] cuisine?” and if yes “How often, on average over the past year, have you ate food from [American, Mexican, Italian, Lebanese, Peruvian, Moroccan, Chinese, Japanese, Greek] style restaurants?” included at the end of Study 1 and Study 2 is provided **Table S1**.

**Table S1.** Level of familiarity for each cuisine (n=1,001)

|  | **Study 1** | | | **Study 2** | | |
| --- | --- | --- | --- | --- | --- | --- |
| **Cuisine** | **Familiarity score^a^**  *Mean* ± *SD* | **Participants that are familiar^b^**  *%* | **Participants that are unfamiliar^c^**  *%* | **Familiarity score^a^**  *Mean* ± *SD* | **Participants that are familiar^b^**  *%* | **Participants that are unfamiliar^c^**  *%* |
| American | 2.84 ± 0.86 | 97.0 | 3.0 | 2.81 ± 0.97 | 94.9 | 5.1 |
| Mexican | 2.42 ± 0.82 | 91.1 | 8.9 | 2.18 ± 0.99 | 81.9 | 18.1 |
| Italian | 2.16 ± 0.78 | 85.9 | 14.1 | 1.99 ± 0.93 | 75.0 | 25.0 |
| Lebanese | 0.50 ± 0.86 | 17.7 | 82.3 |  |  |  |
| Peruvian | 0.38 ± 0.76 | 13.4 | 86.6 |  |  |  |
| Moroccan | 0.41 ± 0.76 | 14.0 | 86.0 |  |  |  |
| Chinese |  |  |  | 2.00 ± 0.94 | 77.2 | 22.8 |
| Japanese |  |  |  | 1.25 ± 1.05 | 43.5 | 56.5 |
| Greek |  |  |  | 1.03 ± 0.99 | 34.2 | 65.8 |

^a^0: never tried; 1: not in the last year; 2: less than once per month; 3: 1-3 times per month; 4: 1-2 times per week; 5: 3 times per week or more. ^b^2 to 5. ^c^0 or 1

1. **Counterbalancing order of dishes**

In order to prevent potential bias due to the order of the dishes on the menu (e.g. participants favouring the first option on the menu), dishes were counterbalanced based on energy content. For the six menus, the lowest and highest energy item were identified and the remaining eight items were categorised as either ‘higher’ or ‘lower’ energy items. **Table S2** shows an example for the American menu in A- conditions.

**Table S2.** Classification of the America menu items (A- conditions)

| **Menu item** | **Energy (kcal)** | **Classification** |
| --- | --- | --- |
| Classic Broccoli Blackened Shrimp Alfredo | 1360 | Highest |
| Riblet Platter | 1290 | Higher |
| Brunch Burger | 1220 | Higher |
| Double Crunch Shrimp | 1170 | Higher |
| Clubhouse Grillle | 1040 | Higher |
| Grilled Chicken Caesar Salad | 970 | Lower |
| Classic Bacon Cheeseburger | 870 | Lower |
| Chicken Wonton Stir Fry | 790 | Lower |
| Signature Bourbon Street Steak | 650 | Lower |
| Cedar Salmon with Maple Mustard Glaze | 370 | Lowest |

The order in which higher and lower calorie items were presented on the main menu was counterbalanced to ensure that the lowest and highest calorie options were not always in the same place on the menu (i.e. towards the top, middle or bottom). **Table S3** outlines how ‘top’, ‘middle’ and ‘bottom’ of the menu were operationalised.

**Table S3.** Description of ‘top’, ‘middle’ and ‘bottom’ positions on the menus

| Position | |
| --- | --- |
| Position 1 | Top |
| Position 2 | Top |
| Position 3 | Middle |
| Position 4 | Middle |
| Position 5 | Middle |
| Position 6 | Middle |
| Position 7 | Middle |
| Position 8 | Middle |
| Position 9 | Bottom |
| Position 10 | Bottom |

When ‘higher’ or ‘lower’ items were featured in either the ‘top’ or ‘bottom’ positions, they were counterbalanced by the opposite pair. **Table S4** shows the six versions of a menu.

**Table S4.** Counterbalanced order of the dishes presented in each main menu

| Position | Version 1 | Version 2 | Version 3 | Version 4 | Version 5 | Version 6 |
| --- | --- | --- | --- | --- | --- | --- |
| Top | **Highest**  Lower | Lower  **Highest** | **Lowest**  Higher | Higher  **Lowest** | Lower  Higher | Higher  Lower |
| Middle | Higher  Lower  Higher  **Lowest**  Higher  Lower | Lower  Higher  Lower  Higher  Lower  Higher | Lower  Higher  Lower  **Highest**  Lower  Higher | Higher  Lower  Higher  Lower  Higher  Lower | Lower  Higher  **Lowest**  Higher  Lower  Higher | Higher  Lower  **Highest**  Lower  Higher  Lower |
| Bottom | Higher  Lower | **Lowest**  Higher | Lower  Higher | **Highes**t  Lower | Lower  **Highest** | Higher  **Lowest** |

To determine in what order the ‘lower’ and ‘higher’ options would feature in the menu, we used the RAND function in Microsoft Excel.

1. **Example menu**

We provide here one version of the main dishes menu for the American cuisine in the A-L+ condition (**Figure S1**); other versions and other menus are available by request.


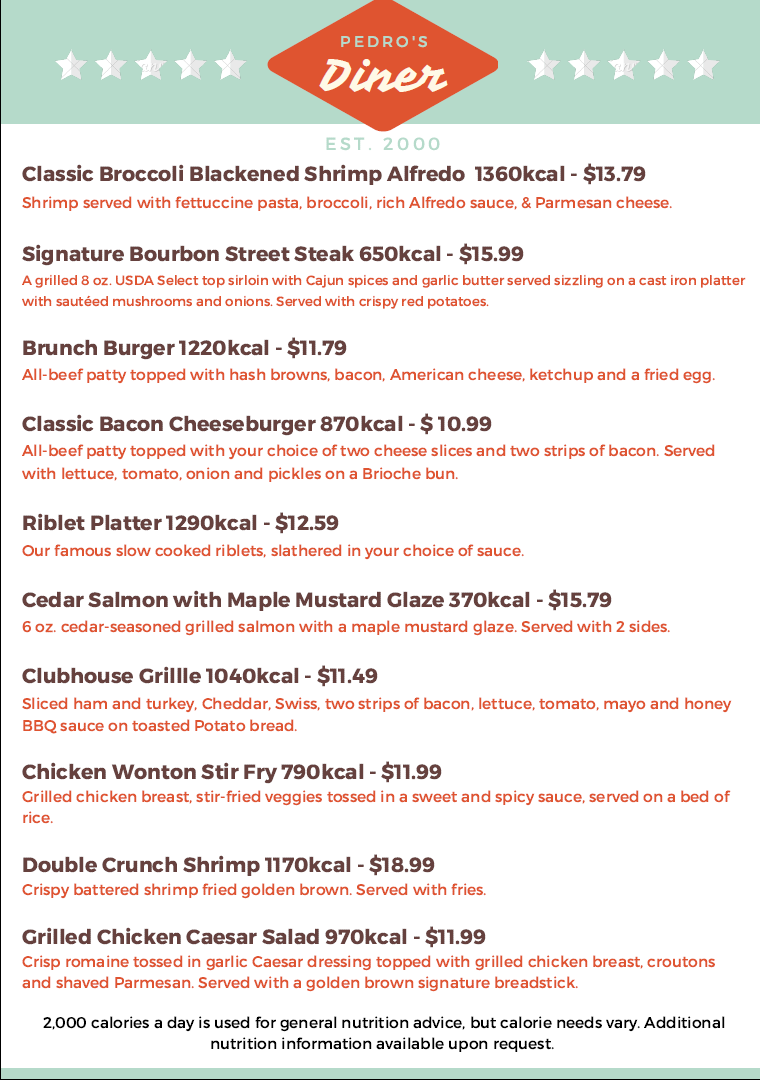


**Figure S1.** American main dishes menu in the A-L+ condition (version 1/6). Menus were created by the research team using Microsoft Powerpoint.

1. **Demographic-based questionnaire**

Participants first completed demographic-based questions detailed in **Table S5**.

**Table S5.** Demographic-based questionnaire

| **Items** | **Options** |
| --- | --- |
| Gender | o Male  o Female  o Other |
| Age | Free text [range 18-99] |
| Ethnicity | o White, non-Hispanic  o Black  o Asian  o Hispanic  o Other |
| What is your current employment status? | o Full or part-time  o Student  o Retired  o Temporary or permanently sick or disabled  o Looking after home/family  o Other unemployed |
| What is your highest educational qualification? If you are a student please select the diploma being studied for. | o Less than high-school  o High-school completion  o Some college or associate degree  o Bachelor’s degree  o Master’s degree  o Doctoral or professional degree |
| After leaving middle school (i.e. after 8th grade), how many further years of higher education did you study for? | Free text |
| What is your annual household income (before tax), including all earners in your household, in dollars (to the nearest $1000)? | Free text [range 0-999999] |
| How many people live at your house, including you? | Free text [range 1-20] |
| How often, on average over the past year, have you had dinner at restaurants? | o Not in the last year  o Less than once per month  o 1-3 times per month  o 1-2 times per week  o 3 times per week or more |
| Are you currently dieting? | o Yes  o No |
| Weight | in kg or lbs |
| Height | in cm or ft and in |

Participants also rated where they believed they are in society from 1 (people who have the least money, least education and the worst jobs or no job) to 10 (people who have the most money, most education and the best jobs) using the MacArthur scale of subjective social status (SSS)^2^.

1. **Restaurants questionnaire**

At the end of Study 1 and Study 2, participants completed four items to assess the validity of the restaurant menus and whether they were influenced by the energy content of food when choosing using a scale from 1: strongly disagree; 2: disagree; 3: slightly disagree; 4: neither agree or disagree; 5: slightly agree; 6: agree; 7: strongly agree (**Table S6**).

**Table S6.** Descriptive analyses for each item of the debriefing questionnaire

| **Items** | **Mean ± SD^a^** | **Disagree^b^ (%)** | **Neutral^c^ (%)** | **Agree^d^**  **(%)** |
| --- | --- | --- | --- | --- |
| *1. There was an acceptable number of food items on the menus.*  Study 1  Study 2 | 5.73 ± 1.22  5.62 ± 1.35 | 8.9  9.5 | 2.5  6.8 | 88.6  83.7 |
| *2. The food choices I made for each dining out scenario were influenced by how many calories I thought were in the food options available.*  Study 1  Study 2 | 2.50 ± 1.68  2.70 ± 1.75 | 76.5  69.5 | 5.3  10.2 | 18.2  20.3 |
| *3. The food items available in the menus are common in (...) style restaurants.* |  |  |  |  |
| *American*  Study 1  Study 2  *Mexican*  Study 1  Study 2  *Italian*  Study 1  Study 2  *Lebanese* – Study 1  *Peruvian* – Study 1  *Moroccan* – Study 1  *Chinese* – Study 2  *Japanese* – Study 2  *Greek* – Study 2 | 6.01 ± 1.13  5.69 ± 1.17  6.05 ± 0.99  5.70 ± 1.17  6.06 ± 0.96  5.76 ±1.09  4.95 ± 1.32  4.92 ± 1.31  4.94 ± 1.32  5.55 ±1.23  5.52 ± 1.20  5.35 ± 1.25 | 5.6  5.9  3.2  5.5  2.2  3.7  6.5  5.9  6.0  7.2  4.1  5.4 | 2.9  7.1  2.7  8.4  4.7  9.1  43.2  44.5  43.0  11.3  18.5  22.5 | 91.5  87.0  94.1  86.1  93.1  87.2  50.3  49.6  51.0  81.5  77.4  72.1 |
| *4. The food items I picked for my meal in the (...) style restaurant would be something that I would normally order in the real world.* |  |  |  |  |
| *American*  Study 1  Study 2  *Mexican*  Study 1  Study 2  *Italian*  Study 1  Study 2  *Lebanese* – Study 1  *Peruvian* – Study 1  *Moroccan* – Study 1  *Chinese* – Study 2  *Japanese* – Study 2  *Greek* – Study 2 | 6.36 ± 0.95  6.14 ± 1.06  6.22 ± 1.09  5.90 ± 1.29  6.29 ± 0.98  6.03 ± 1.08  5.38 ± 1.61  5.38 ± 1.63  5.38 ± 1.63  5.83 ± 1.32  5.66 ± 1.46  5.69 ± 1.37 | 2.5  3.6  3.6  6.3  2.2  3.4  12.5  13.4  13.3  6.8  8.8  6.8 | 1.0  3.6  2.4  5.7  2.6  6.1  14.4  13.9  13.6  7.9  11.5  13.0 | 96.5  92.8  94.0  88.0  95.2  90.5  73.1  72.7  73.1  85.3  79.7  80.2 |

^a^Range: 1: strongly disagree; 2: disagree; 3: slightly disagree; 4: neither agree or disagree; 5: slightly agree; 6: agree; 7: strongly agree. ^b^1 to 3. ^c^4. ^d^5 to 7.

ANOVA models were used to test the effect of availability, labelling and highest educational level on each item of the debriefing questionnaire to investigate whether the responses differed across experimental conditions and educational level (**Table S7**).

**Table S7.** ANOVA models, dependant variables: debriefing questionnaire items

| **Model** | ***F*** | ***p*** | **partial *η^2^*** |
| --- | --- | --- | --- |
| **Item 1 - Study 1**  Availability  Labelling  Education  **Item 1 - Study 2**  Availability  Labelling  Education | 0.05  0.02  0.24  0.31  2.79  0.02 | 0.829  0.876  0.624  0.577  0.095  0.901 | < 0.001  < 0.001  < 0.001  < 0.001  0.003  < 0.001 |
| **Item 2 – Study 1**  Availability  Labelling  Education  **Item 2 – Study 2**  Availability  Labelling  Education | 0.09  1.94  14.46  0.19  17.13  17.71 | 0.764  0.164  < 0.001  0.664  < 0.001  < 0.001 | < 0.001  0.002  0.014  < 0.001  0.016  0.016 |
| **Item 3 – Study 1**  ***Familiar***  Availability  Labelling  Education  ***Unfamiliar***  Availability  Labelling  Education | 1.67  0.26  1.01  0.52  0.10  9.34 | 0.197  0.609  0.315  0.471  0.757  0.002 | 0.002  < 0.001  0.001  0.001  < 0.001  0.009 |
| **Item 3 – Study 2**  ***All cuisines***  Availability  Labelling  Education | 4.62  0.17  0.04 | 0.032  0.678  0.840 | 0.004  < 0.001  < 0.001 |
| **Item 4 – Study 1**  ***Familiar***  Availability  Labelling  Education  ***Unfamiliar***  Availability  Labelling  Education | 0.15  0.55  1.22  0.06  0.03  8.55 | 0.698  0.458  0.269  0.814  0.870  0.004 | < 0.001  0.001  0.001  < 0.001  < 0.001  0.009 |
| **Item 4 – Study 2**  ***All cuisines***  Availability  Labelling  Education | 0.05  0.92  0.34 | 0.822  0.339  0.560 | < 0.001  0.001  < 0.001 |

Summary of main significant ANOVA findings in table S7: In Study 2, participants reported that their food choices were influenced by energy content to a larger extent in the labelling condition compared to the no labelling condition. In both studies, participants of higher educational qualification reported that their food choices were more influenced by energy content than participants from lower educational qualification. There were no other significant differences.

1. **Alternative continuous measure of education level**

A continuous measure of education level was used in sensitivity analyses: the composite score ‘level of education’ calculated as the average of the z-scores of highest educational qualification (coded from 1 = less than high school to 6 = doctoral or professional degree) and years in higher education.

1. **Power calculation**

Based on a previous trial using a virtual fast food restaurant, we estimated a between-subject standard deviation of 217.3 kcal for meal orders. Simulations using MLPowSim Software Package^3^ showed that a sample size of 1,000 participants or more with α = 0.05 allows the detection of a 2% energy reduction due to labelling or availability, and an additional 2% energy reduction due to an interaction between labelling and highest educational qualification, at power > 0.80. Empirical estimates of sample sizes needed for 0.80 power in mediation analyses indicate that samples of ≈ 380 are sufficient to detect mediation through pathways that are small and small-to-medium in statistical size using bias-corrected bootstrap tests^4^. Thus, a sample of 1,000 participants for each study allowed for adequate power for moderated mediation analysis.

1. **Participants characteristics overall and across experimental conditions**

**Table S8.** Study 1 participants characteristics

|  | **All**  **(n=1001)** | **Baseline availability &**  **No labelling**  **(n=250)** | **Baseline availability &**  **Labelling**  **(n=254)** | **Increased availability &**  **No labelling**  **(n=247)** | **Increased availability &**  **Labelling**  **(n=250)** |
| --- | --- | --- | --- | --- | --- |
| **Age, years, mean (SD)** | 35.3 (12.7) | 34.0 (11.9) | 36.5 (13.4) | 36.0 (13.0) | 34.6 (12.2) |
| **Gender, n (%)**  *Male*  *Female*  *Other* | 469 (46.8)  532 (53.2)  0 (0) | 123 (49.2)  127 (50.8)  0 (0) | 117 (46.1)  137 (53.9)  0 (0) | 123 (49.8)  124 (50.2)  0 (0) | 106 (42.4)  144 (57.6)  0 (0) |
| **Ethnicity, n (%)**  *White, non-hispanic*  *Black*  *Asian*  *Hispanic*  *Other* | 772 (77.1)  78 (7.8)  44 (4.4)  77 (7.7)  30 (3.0) | 194 (77.6)  21 (8.4)  9 (3.6)  21 (8.4)  5 (2.0) | 195 (76.8)  15 (5.9)  12 (4.7)  24 (9.5)  8 (3.1) | 193 (78.1)  17 (6.9)  12 (4.9)  16 (6.5)  9 (3.6) | 190 (76.0)  25 (10.0)  11 (4.4)  16 (6.4)  8 (3.2) |
| **BMI, kg/m^2^, mean (SD)**  *Missing or implausible^a^, n (%)* | 28.2 (7.75)  79 (7.9) | 27.8 (6.94)  23 (9.2) | 28.6 (8.45)  16 (6.3) | 27.7 (7.77)  22 (8.9) | 28.5 (7.73)  18 (7.2) |
| **Employment status, n (%)**  *Full or part-time*  *Student*  *Retired*  *Sick or disabled*  *Looking after home*  *Other unemployed* | 606 (60.5)  78 (7.8)  36 (3.6)  30 (3.0)  122 (12.2)  129 (12.9) | 159 (63.6)  22 (8.8)  7 (2.8)  6 (2.4)  29 (11.6)  27 (10.8) | 157 (61.8)  16 (6.3)  7 (2.8)  8 (3.1)  32 (12.6)  34 (13.4) | 149 (60.3)  21 (8.5)  9 (3.6)  9 (3.6)  30 (12.2)  29 (11.8) | 141 (56.4)  19 (7.6)  13 (5.2)  7 (2.8)  31 (12.4)  39 (15.6) |
| **Highest educational level, n (%)**  *Less than high-school*  *High-school completion*  *Some college or associate degree*  *Bachelor’s degree*  *Master’s degree*  *Doctoral or professional degree* | 26 (2.6)  325 (32.5)  365 (36.5)  199 (19.9)  59 (5.9)  27 (2.7) | 5 (2.0)  82 (32.8)  88 (35.2)  57 (22.8)  15 (6.0)  3 (1.2) | 7 (2.7)  80 (31.5)  99 (39.0)  46 (18.1)  18 (7.1)  4 (1.6) | 7 (2.8)  79 (32.0)  88 (35.7)  55 (22.3)  9 (3.6)  9 (3.6) | 7 (2.8)  84 (33.6)  90 (36.0)  41 (16.4)  17 (6.8)  11 (4.4) |
| **Highest educational level binary, n (%)**  *Low (≤ High-school completion)*  *High (> High-school completion)* | 351 (35.1)  650 (64.9) | 87 (34.8)  163 (65.2) | 87 (34.3)  167 (65.7) | 86 (34.8)  161 (65.2) | 91 (36.4)  159 (63.6) |
| **Years of higher education, mean (SD)** | 5.88 (2.37) | 5.84 (2.31) | 5.88 (2.34) | 5.91 (2.34) | 5.87 (2.50) |
| **Household income, $, mean (SD)** | 56432  (45831) | 57041  (51345) | 57164  (42726) | 53777  (42332) | 57704  (46526) |
| **Subjective socioeconomic status, mean (SD)^b^** | 4.68 (1.75) | 4.54 (1.72) | 4.72 (1.72) | 4.83 (1.83) | 4.64 (1.71) |
| **Dining out frequency, n (%)**  Not in the last year  Less than once per month  1-3 times per month  1-2 times per week  3 times per week or more | 30 (3.0)  268 (26.8)  486 (48.5)  172 (17.2)  45 (4.5) | 7 (2.8)  63 (25.2)  129 (51.6)  36 (14.4)  15 (6.0) | 12 (4.7)  58 (22.9)  123 (48.4)  49 (19.3)  12 (4.7) | 6 (2.4)  75 (30.4)  117 (47.4)  38 (15.4)  11 (4.4) | 5 (2.0)  72 (28.8)  117 (46.8)  49 (19.6)  7 (2.8) |
| **Dieting status, yes, n (%)** | 138 (13.8) | 31 (12.4) | 36 (14.2) | 37 (15.0) | 34 (13.6) |

^a^Self-reported body mass index (BMI) was calculated in kg/m^2^. BMI implausible values: BMI>10 or BMI<60 ^5^ ^b^Higher scores indicate higher perceived socioeconomic status, scale range 1-10

**Table S9.** Study 2 participants characteristics

|  | **All**  **(n=1090)** | **Baseline availability &**  **No labelling**  **(n=275)** | **Baseline availability &**  **Labelling**  **(n=272)** | **Increased availability &**  **No labelling**  **(n=271)** | **Increased availability &**  **Labelling**  **(n=272)** |
| --- | --- | --- | --- | --- | --- |
| **Age, years, mean (SD)** | 44.9 (18.5) | 46.3 (18.9) | 44.0 (17.6) | 44.1(18.8) | 45.2 (18.6) |
| **Gender, n (%)** |  |  |  |  |  |
| *Male*  *Female*  *Other*  **Ethnicity, n (%)**  *White, non-hispanic*  *Black*  *Asian*  *Hispanic*  *Other* | 453 (41.6)  624 (57.2)  13 (1.2)  916 (84.0)  76 (7.0)  27 (2.5)  45 (4.1)  26 (2.4) | 113 (41.1)  159 (57.8)  3 (1.1)  230 (83.6)  20 (7.3)  8 (2.9)  11 (4.0)  6 (2.2) | 120 (44.1)  149 (54.8)  3 (1.1)  228 (83.9)  22 (8.1)  6 (2.2)  8 (2.9)  8 (2.9) | 102 (37.6)  165 (60.9)  4 (1.5)  230 (84.9)  17 (6.3)  5 (1.8)  13 (4.8)  6 (2.2) | 118 (43.4)  151 (55.5)  3 (1.1)  228 (83.8)  17 (6.3)  8 (2.9)  13 (4.8)  6 (2.2) |
| **BMI, kg/m^2^, mean (SD)**  *Missing or implausible^a^, n (%)* | 28.1 (7.43)  20 (1.8) | 27.8 (6.96)  1 (0.6) | 27.90 (7.20)  6 (2.2) | 28.9 (8.06)  3 (1.1) | 27.8 (7.45)  10 (3.7) |
| **Employment status, n (%)**  *Full or part-time*  *Student*  *Retired*  *Sick or disabled*  *Looking after home*  *Other unemployed* | 482 (44.2)  57 (5.2)  248 (22.7)  75 (6.9)  127 (11.7)  101 (9.3) | 123 (44.7)  10 (3.7)  69 (25.1)  19 (6.9)  27 (9.8)  27 (9.8) | 136 (50.0)  15 (5.5)  58 (21.3)  17 (6.3)  22 (8.1)  24 (8.8) | 114 (42.1)  14 (5.1)  62 (22.9)  17 (6.3)  42 (15.5)  22 (8.1) | 109 (40.1)  18 (6.6)  59 (21.7)  22 (8.1)  36 (13.2)  28 (10.3) |
| **Highest educational qualification, n (%)**  *Less than high-school*  *High-school completion*  *Some college or associate degree*  *Bachelor’s degree*  *Master’s degree*  *Doctoral or professional degree* | 75 (6.9)  313 (28.7)  365 (33.5)  231 (21.2)  92 (8.4)  14 (1.3) | 21 (7.6)  80 (29.1)  83 (30.2)  63 (22.9)  26 (9.5)  2 (0.7) | 19 (7.0)  70 (25.7)  98 (36.0)  57 (21.0)  22 (8.1)  6 (2.2) | 20 (7.4)  87 (32.1)  90 (33.2)  50 (18.5)  21 (7.7)  3 (1.1) | 15 (5.5)  76 (27.9)  94 (34.6)  61 (22.4)  23 (8.5)  3 (1.1) |
| **Highest educational qualification binary, n (%)**  *Low (≤ High-school completion)*  *High (> High-school completion)* | 388 (35.6)  702 (64.4) | 101 (36.7)  174 (63.3) | 89 (32.7)  183 (67.3) | 107 (39.5)  164 (60.5) | 91 (33.5)  181 (66.5) |
| **Years of higher education, mean (SD)** | 5.90 (2.57) | 5.87 (2.56) | 6.03 (2.58) | 5.72 (2.54) | 5.99 (2.62) |
| **Household income, $, mean (SD)** | 53165 (49129) | 57033 (48725) | 51587 (48145) | 48551 (39464) | 55432 (58183) |
| **Subjective socioeconomic status, mean (SD)^b^** | 4.98 (1.88) | 5.02 (1.92) | 4.99 (1.90) | 4.92 (1.86) | 4.99 (1.86) |
| **Dining out frequency, n (%)**  Not in the last year  Less than once per month  1-3 times per month  1-2 times per week  3 times per week or more | 55 (5.0)  323 (29.6)  473 (43.4)  186 (17.1)  53 (4.9) | 6 (2.2)  82 (29.8)  136 (49.5)  44 (16.0)  7 (2.5) | 13 (4.8)  73 (26.8)  130 (47.8)  42 (15.4)  14 (5.2) | 18 (6.6)  82 (30.3)  105 (38.7)  52 (19.2)  14 (5.2) | 18 (6.6)  86 (31.6)  102 (37.5)  48 (17.7)  18 (6.6) |
| **Dieting status, yes, n (%)** | 116 (10.6) | 30 (10.9) | 23 (8.5) | 29 (10.7) | 34 (12.5) |

^a^Self-reported body mass index (BMI) was calculated in kg/m^2^. BMI implausible values: BMI>10 or BMI<60 ^5^ ^b^Higher scores indicate higher perceived socioeconomic status, scale range 1-10

1. **Effect of the interventions and cuisine familiarity (Study 1)**

One of the secondary aims of the Study 1 was to examine the effect of the interventions on familiar and unfamiliar cuisines menus (e.g. is intervention effectiveness moderated by cuisine familiarity). A linear mixed model was used to test the effect of labelling, availability, highest educational qualification, familiarity and the following interactions: labelling* highest educational qualification, availability* highest educational qualification, labelling*familiarity, availability*familiarity with participant effect and menu effect as random. Familiarity did not significantly moderate the effect of either intervention (secondary analyses, *p* < 0.01) in Study 1 (**Table S10)**, indicating that the effects of energy labelling and increasing availability of lower energy menu options were similar for familiar and less familiar cuisine menus.

**Table S10.** Fixed effect statistics of complete and stratified linear mixed models with participant and menu effect as random, dependant variable: energy of the main.

|  | **Type III tests** | | ***Estimate^a^*** | ***95% LCL*** | ***95% UCL*** |
| --- | --- | --- | --- | --- | --- |
| **Model** | ***F*** | ***p*** |  |  |  |
| **Complete (n=1001)**  (Intercept)  Availability  Labelling  Education  Familiarity  Availability*Education  Labelling*Education  Availability*Familiarity  Labelling*Familiarity | 256.43  9.25  1.62  0.12  < 0.01  6.78  2.28  3.89 | < 0.001  0.002  0.203  0.730  0.968  0.009  0.131  0.049 | 979.74  -118.15  -16.92  11.00  -35.09  -0.64  -41.85  -20.52  26.79 | 795.02  -146.83  -45.60  -16.34  -217.63  -32.16  -73.37  -47.15  0.16 | 1164.47  -89.47  11.76  38.33  147.44  30.88  -10.33  6.11  53.43 |
| **Education = ‘low’ (n=351)**  (Intercept)  Availability  Labelling  Familiarity  Availability*Familiarity  Labelling*Familiarity | 105.86  0.08  0.10  0.37  1.09 | < 0.001  0.778  0.753  0.541  0.296 | 979.60  -121.49  -15.35  -34.81  -13.84  23.65 | 789.91  -154.54  -48.40  -223.90  -58.24  -20.75 | 1169.30  -88.45  17.70  154.28  30.57  68.06 |
| **Education = ‘high’ (n=650)**  (Intercept)  Availability  Labelling  Familiarity  Availability*Familiarity  Labelling*Familiarity | 176.88  21.87  0.13  2.02  2.79 | < 0.001  < 0.001  0.718  0.155  0.095 | 990.80  -116.98  -59.57  -35.21  -24.14  28.38 | 808.98  -142.27  -84.85  -216.45  -57.45  -4.92 | 1172.62  -91.70  -34.29  146.02  9.17  61.69 |

^a^Intercept estimate for the reference group: baseline availability, no labelling, low education level (complete model), unfamiliar cuisine; estimates of the fixed effects must be interpreted as differences with the reference group.

1. **Sensitivity analyses**
   1. *Excluding aim guessers*

In Study 1, 52 participants identified the aim of the study. In Study 2, 18 participants identified the study aims. Linear mixed models were re-run with aim guessers excluded (**Table S11**). The pattern of results remained the same as in the main analyses for both Study 1 and Study 2.

**Table S11.** Fixed effect statistics of complete and stratified linear mixed models without aim guessers with participant and menu effect as random, dependant variable: energy of the main.

|  | **Study 1** | | | | |
| --- | --- | --- | --- | --- | --- |
|  | **Type III effects** | | ***Estimate^a^*** | ***95% LCL*** | ***95% UCL*** |
| **Model** | ***F*** | ***p*** |  |  |  |
| **Complete (n=949)**  (Intercept)  Availability  Labelling  Education  Availability*Education  Labelling*Education | 265.94  7.75  0.43  < 0.01  3.92 | < 0.001  0.005  0.510  0.959  0.048 | 963.63  -131.30  -6.49  11.10  -0.83  -32.02 | 851.00  -156.62  -31.82  -15.99  -32.50  -63.71 | 1076.26  -105.98  18.83  38.19  30.84  -0.33 |
| **Education = ‘low’ (n=342)**  (Intercept)  Availability  Labelling | 108.21  0.26 | < 0.001  0.607 | 963.63  -131.30  -6.49 | 850.01  -156.06  -31.25 | 1077.26  -106.54  18.26 |
| **Education = ‘high’ (n=607)**  Intercept  Availability  Labelling | 180.97  15.32 | < 0.001  < 0.001 | 974.73  -132.13  -38.51 | 864.53  -151.39  -57.80 | 1084.94  -112.87  -19.22 |
|  | **Study 2** | | | | |
|  | **Type III effects** | | ***Estimate*** | ***95% LCL*** | ***95% UCL*** |
| **Model** | ***F*** | ***p*** |  |  |  |
| **Complete (n=1072)**  (Intercept)  Availability  Labelling  Education  Availability*Education  Labelling*Education | 238.90  8.39  2.11  1.74  3.49 | < 0.001  0.004  0.146  0.187  0.062 | 957.53  -115.20  -8.40  14.15  -21.53  -30.49 | 917.97  -140.82  -34.09  -13.21  -53.48  -62.50 | 997.09  -89.58  17.29  41.50  10.43  1.52 |
| **Education = ‘low’ (n=383)**  (Intercept)  Availability  Labelling | 81.54  0.43 | < 0.001  0.512 | 957.53  -115.20  -8.40 | 923.65  -140.22  -33.49 | 991.42  -90.18  16.69 |
| **Education = ‘high’ (n=689)**  (Intercept)  Availability  Labelling | 191.96  15.53 | < 0.001  < 0.001 | 971.68  -136.73  -38.89 | 932.77  -156.08  -58.24 | 1010.58  -117.38  -19.54 |

^a^Intercept estimate for the reference group: baseline availability, no labelling, low education level (complete models); estimates of the fixed effects must be interpreted as differences with the reference group.

- 1. *Substituting the categorical variable ‘highest educational qualification’ by the continuous composite variable ‘level of education’*

Highest educational qualification and years in higher education were measured because both educational achievements time spent in education may be important in determining SEP^6^. In addition, SEP has been defined as a hierarchical series of layers^7^ and thus a continuous variable might match this definition more closely than a binary variable. In both Study 1 and Study 2, the composite score ‘level of education’ was calculated as the average of the z-scores of the two variables: ‘highest educational qualification’ (coded from 1 = less than high school; 2 = high school completion; 3 = some college or associate degree; 4 = bachelorˈs degree; 5 = masterˈs degree; 6 = doctoral or professional degree) and ‘years in higher education’ (continuous). Linear mixed models were run to test the effect of the intervention and level of education (**Table S12**). The pattern of results remained the same as when testing the effect of the interventions and highest educational qualification.

**Table S12.** Fixed effect statistics of complete and stratified linear mixed models with the composite score ‘level of education’ with participant and menu effect as random, dependant variable: energy of the main.

|  | **Study 1** | | | | |
| --- | --- | --- | --- | --- | --- |
|  | **Type III effects** | | ***Estimate^a^*** | ***95% LCL*** | ***95% UCL*** |
| **Model** | ***F*** | ***p*** |  |  |  |
| **Complete (n=1001)**  (Intercept)  Availability  Labelling  Level of education  Availability*Level of education  Labelling*Level of education | 280.81  16.05  7.94  0.11  3.68 | < 0.001  < 0.001  0.005  0.738  0.055 | 969.12  -128.30  -30.67  -2.26  -2.66  -15.22 | 860.11  -143.30  -45.68  -16.19  -18.22  -30.76 | 1078.13  -113.29  -15.66  11.67  12.90  0.32 |
| **Level of education < 0 (n=496)**^b^  (Intercept)  Availability  Labelling | 135.96  0.39 | < 0.001  0.534 | 963.71  -125.38  -6.68 | 854.13  -146.47  -27.75 | 1073.30  -104.30  14.40 |
| **Level of education ≥ 0 (n=505)**  (Intercept)  Availability  Labelling | 149.19  25.20 | < 0.001  < 0.001 | 975.71  -133.28  -54.77 | 863.83  -154.67  -76.16 | 1087.60  -111.88  -33.37 |
|  | **Study 2** | | | | |
|  | **Type III effects** | | ***Estimate*** | ***95% LCL*** | ***95% UCL*** |
| **Model** | ***F*** | ***p*** |  |  |  |
| **Complete (n=1090)**  (Intercept)  Availability  Labelling  Level of education  Availability*Level of education  Labelling*Level of education | 279.11  11.55  1.78  1.44  2.31 | < 0.001  < 0.001  0.182  0.230  0.128 | 966.86  -129.82  -26.42  5.63  -9.75  -12.37 | 934.40  -145.05  -41.67  -8.14  -25.69  -28.32 | 999.32  -114.59  -11.18  19.40  6.18  3.57 |
| **Level of education < 0 (n=519)**  (Intercept)  Availability  Labelling | 111.53  3.15 | < 0.001  0.076 | 959.97  -114.78  -19.32 | 927.50  -136.09  -40.65 | 992.43  -93.47  2.01 |
| **Level of education ≥ 0 (n=571)**  (Intercept)  Availability  Labelling | 169.08  9.27 | < 0.001  0.002 | 973.43  -143.83  -33.69 | 929.30  -165.52  -55.39 | 1017.56  -122.14  -12.00 |

^a^Intercept estimate for the reference group: baseline availability, no labelling; estimates of the fixed effects must be interpreted as differences with the reference group. ^b^The composite score ‘level of education’ is centred on 0

1. **Bayesian analyses on pooled data**

Bayes factors (denoted as ‘BF_10_’ and ‘BF_01_’) quantify evidence for each alternative model over a reference model – which can be the null model or another model chosen as reference. BF_10_=x means that the alternative model is x times more plausible than the reference model given the data, whereas BF_01_=1/BF_10_=y means that the reference model is y times more plausible than the alternative model. Bayes factors are continuous, however discrete categories have been suggested to aid interpretation. According to Raftery’s classification^8^, a Bayes factor of 1-3 indicates ‘weak support’, 3-20 ‘positive support’, 20-150 ‘strong support’, and >150 ‘very strong support’. Bayes factors were calculated using JASP 0.9.2 and using a default prior r scale of 0.707. Because Bayesian models with repeated measures did not converge, repeated menu ordering was aggregated at participant level by calculating the average energy of the main chosen for all of the six cuisines and Bayesian ANOVA was performed.

**Table S13.** Bayes factors for comparison of the likelihood of energy of the main pooled data under several models

| Models | BF_10_ | BF_01_ | Raftery’s classification |
| --- | --- | --- | --- |
| **Reference (H0):** Null model  **Alternatives (H1):**  + availability  + labelling  + highest educational qualification | 2.343e+104  1979  0.107 | 4.267e-105  5.052e-4  9.310 | Very strong support for H1  Very strong support for H1  Positive support for H0 |
| **Reference (H0):** availability + labelling + highest educational qualification  **Alternatives (H1):**  + availability* highest educational qualification  + labelling* highest educational qualification | 0.100  8.088 | 10.019  0.124 | Positive support for H0  Positive support for H1 |

Bayes factors analysis confirmed the results obtained with frequentist statistical methods: availability and labelling had a strong effect on energy of the main ordered and a model including labelling* highest educational qualification interaction was more likely than the complete model without interaction.

1. **Effect of the interventions on energy content of all dishes ordered (pooled data)**

**Table S14.** Fixed effect statistics of complete and stratified linear mixed models with participant and menu effect as random, dependant variable: total energy ordered (mains, sides and desserts)

|  | **Type III effects** | | ***Estimate^a^*** | ***95% LCL*** | ***95% UCL*** |
| --- | --- | --- | --- | --- | --- |
| **Model** | ***F*** | ***p*** |  |  |  |
| **Complete (n=2,091)**  (Intercept)  Availability  Labelling  Education  Availability*Education  Labelling*Education  Study | 36.79  17.43  21.04  0.03  < 0.01  3.41 | < 0.001  < 0.001  < 0.001  0.873  0.981  0.065 | 1764.18  -114.14  -80.27  -85.13  -6.19  -0.92  -37.69 | 1595.20  -175.07  -141.24  -150.54  -81.96  -76.74  -77.68 | 1933.16  -53.21  -19.30  -19.72  69.59  74.91  2.31 |
| **Education = ‘low’ (n=739)**  (Intercept)  Availability  Labelling  Study | 13.50  6.31  1.06 | < 0.001  0.012  0.304 | 1754.68  -114.41  -78.33  -35.16 | 1564.72  -175.46  -139.46  -102.21 | 1944.64  -53.36  -17.21  31.89 |
| **Education = ‘high’ (n=1352)**  (Intercept)  Availability  Labelling  Study | 27.52  12.39  2.48 | < 0.001  < 0.001  0.115 | 1684.00  -120.35  -80.77  -39.97 | 1525.57  -165.32  -125.75  -89.69 | 1842.43  -75.38  -35.79  9.75 |

^a^Intercept estimate for the reference group: baseline availability, no labelling, low education level (complete model), study 1; estimates of the fixed effects must be interpreted as differences with the reference group.

1. **Effect of the interventions on energy content of extra dishes ordered only – sides and desserts (pooled data)**

**Table S15.** Fixed effect statistics of complete and stratified linear mixed models with participant and menu effect as random, dependant variable: extra energy ordered (sides and desserts) *–* only orders with extra sides or extra desserts

|  | **Type III effects** | | ***Estimate^a^*** | ***95% LCL*** | ***95% UCL*** |
| --- | --- | --- | --- | --- | --- |
| **Model** | ***F*** | ***p*** |  |  |  |
| **Complete (n=2060)**  (Intercept)  Availability  Labelling  Education  Availability*Education  Labelling*Education  Study | 0.36  14.87  9.26  0.51  0.12  0.01 | 0.548  < 0.001  0.002  0.476  0.727  0.929 | 841.33  -1.76  -66.21  -64.64  22.43  10.98  -1.49 | 664.05  -51.12  -115.62  -117.90  -39.28  -50.78  -34.46 | 1018.61  47.61  -16.80  -11.39  84.13  72.75  31.48 |
| **Education = ‘low’ (n=734)**  (Intercept)  Availability  Labelling  Study | < 0.01  6.00  0.13 | 0.95  0.01  0.72 | 826.17  -1.58  -64.63  10.44 | 639.75  -53.22  -116.35  -46.33 | 1012.59  50.06  -12.92  67.20 |
| **Education = ‘high’ (n=1326)**  (Intercept)  Availability  Labelling  Study | 1.27  8.90  0.17 | 0.260  0.003  0.679 | 784.14  20.73  -54.89  -8.54 | 612.22  -15.32  -90.96  -48.93 | 956.05  56.78  -18.82  31.86 |

^a^Intercept estimate for the reference group: baseline availability, no labelling, low education level (complete model), study 1; estimates of the fixed effects must be interpreted as differences with the reference group.

1. **Moderated mediation analyses**

Moderated mediation analysis was performed in an attempt to identify why energy labelling resulted in a decrease in energy of main ordered in participants of higher, but not lower education level. Specifically, we examined whether 1/ health motivation (Study 1 and Study 2), 2/ weight control motivation (Study 1 and Study 2), 3/ general nutrition knowledge (Study 2)^[[1]](#footnote-1)^, 4/ trait impulsivity (Study 2) and 5/ discount rate (Study 2) mediated the effect of highest educational level on the energy of the main ordered with and without labelling (**Figure S2**). Repeated ordering was aggregated at participant level by calculating the average energy of the main for all of the six cuisines. Conditional indirect effects were calculated using bias-corrected bootstrap (5,000 bootstrap samples) using PROCESS macro version 3 on SAS 9.3 (**Table S16**).


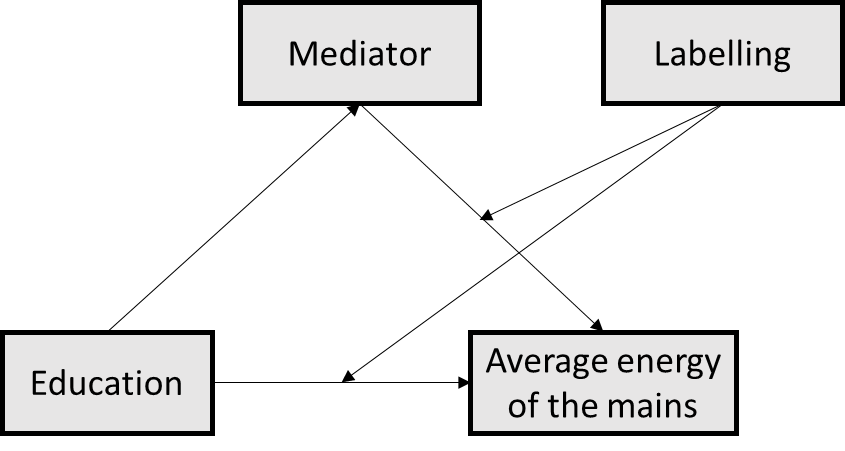


**Figure S2.** Moderated mediation conceptual diagram

**Table S16.** Conditional direct and indirect effects of education on average energy of main menu choice

| **Mediator** | **No labelling** | | | **Labelling** | | |  | | |
| --- | --- | --- | --- | --- | --- | --- | --- | --- | --- |
|  | **Estimate** | **99% LCL** | **99% UCL** | **Estimate** | **99% LCL** | **99% UCL** | **Diff**  **indirect** | **99% LCL** | **99% UCL** |
| **Health motivation – 1**  Indirect effect  Direct effect | **-6.45**  13.66 | **-13.7**  -19.18 | **-1.54**  46.50 | **-7.04**  -17.38 | **-15.00**  -50.47 | **-1.68**  15.70 | -0.59 | -7.49 | 6.20 |
| **Weight control motivation – 1**  Indirect effect  Direct effect | -2.31  12.97 | -7.53  -20.23 | 0.55  46.17 | **-5.01**  -23.06 | **-11.67**  -55.95 | **-0.56**  9.82 | -2.70 | -9.28 | 1.88 |
| **Health motivation – 2**  Indirect effect  Direct effect | -1.92  9.44 | -7.38  -23.18 | 2.90  42.06 | **-11.01**  -15.61 | **-19.84**  -49.32 | **-4.32**  18.10 | **-9.09** | **-18.31** | **-1.56** |
| **Weight control motivation – 2**  Indirect effect  Direct effect | 1.23  6.57 | -2.18  -25.68 | 5.43  38.82 | **-8.10**  -16.15 | **-16.55**  -49.67 | **-1.89**  17.36 | **-9.33** | **-20.11** | **-2.02** |
| **General nutrition knowledge – 2**  Indirect effect  Direct effect | 4.03  3.67 | -2.93  -29.72 | 12.02  37.06 | -5.87  -19.96 | -14.24  -54.62 | 2.10  14.71 | -9.90 | -22.04 | 0.94 |
| **Impulsivity – 2**  Indirect effect  Direct effect | 3.78  4.04 | -2.46  -29.09 | 10.82  37.17 | -5.31  -20.39 | -13.05  -54.81 | 0.95  14.04 | **-9.09** | **-20.07** | **-0.18** |
| **Discount rate – 2**  Indirect effect  Direct effect | -0.13  7.72 | -1.62  -25.01 | 0.89  40.44 | -0.30  -26.16 | -2.32  -59.96 | 1.10  7.64 | -0.17 | -2.45 | 1.66 |

1: measured in Study 1. 2: Measured in Study 2.

In Study 1, no evidence of moderated mediation was found, i.e. the differences between the indirect effects in no labelling vs. labelling condition were not significantly different from zero. In Study 2, the indirect effect of educational qualification on average energy of main menu choice through three mediators (examined separately from each other) were significantly moderated by labelling: health motivation, weight control motivation and impulsivity. The correlation matrix of these three mediators is shown **Table S17**.

**Table S17.** Pearson’s correlations between health motivation, weight control motivation and impulsivity scores adjusted for educational qualification (n=1,090) in Study 2

|  | **Health motivation** | **Weight control motivation** | **Impulsivity** |
| --- | --- | --- | --- |
| Health motivation | - | *r* = 0.62  *p* < 0.001 | *r* = -0.19  *p* < 0.001 |
| Weight control motivation |  | - | \| *r* = -0.08 \| \| --- \| \| *p* = 0.008 \| |
| Impulsivity |  |  | - |

Because the three identified mediators were not independent, we considered all mediators jointly in the same model to control for the fact that they affect one another^9^ (**Figure S3**).


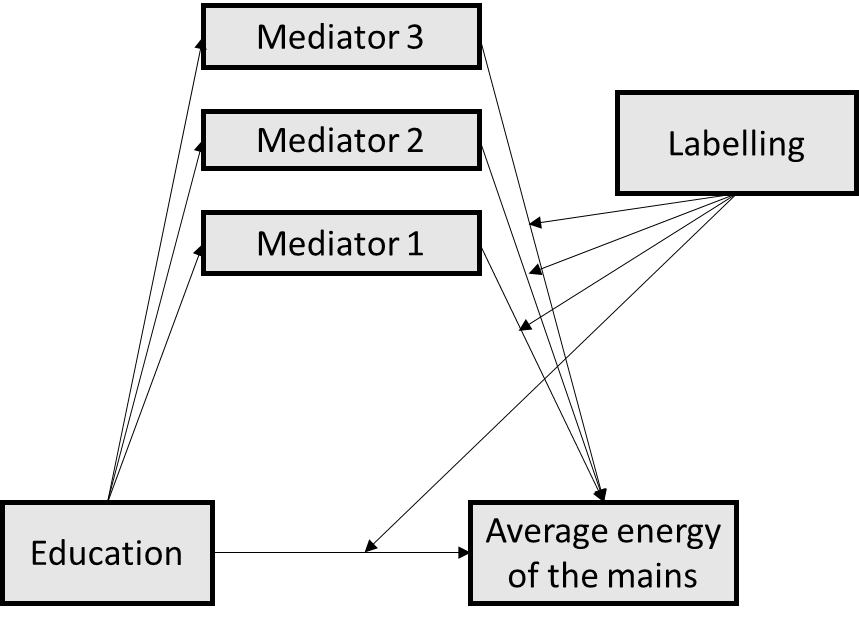


**Figure S3.** Moderated mediation with three mediators conceptual diagram

Conditional indirect effects of the joint model were calculated using bias-corrected bootstrap (5,000 bootstrap samples) using PROCESS macro version 3 on SAS 9.3 (**Table S18**).

**Table S18.** Conditional direct and indirect effects of education on average energy of main menu choice in a joint model including three mediators: health motivation, weight control motivation and impulsivity.

|  | **No labelling** | | | | **Labelling** | | |  | | |
| --- | --- | --- | --- | --- | --- | --- | --- | --- | --- | --- |
|  | **Estimate** | | **99% LCL** | **99% UCL** | **Estimate** | **99% LCL** | **99% UCL** | **Diff**  **indirect** | **99% LCL** | **99% UCL** |
| Indirect effects  *Health*  *Weight control*  *Impulsivity* | -5.83  3.37  4.97 | -14.08  -0.99  -1.59 | | 0.38  9.63  12.94 | -4.92  **-5.85**  -3.84 | -13.40  **-13.54**  -11.14 | 2.19  **-0.67**  2.56 | 0.92  **-9.22**  -8.81 | **-**9.01  **-20.76**  -20.15 | 11.35  **-1.38**  0.29 |
| Direct effect | 6.26 | -26.46 | | 38.98 | -10.00 | -44.07 | 24.07 |  |  |  |

In the joint moderated mediation model, only the indirect effect of educational qualification on average energy of the main menu choice through weight control motivation was significantly moderated by labelling.

**References**

1. Smith M. Italian cuisine is world’s most popular. YouGov. https://yougov.co.uk/topics/food/articles-reports/2019/03/12/italian-cuisine-worlds-most-popular. Published 2019. Accessed June 9, 2018.

2. Adler NE, Epel ES, Castellazzo G, Ickovics JR. Relationship of Subjective and Objective Social Status With Psychological and Physiological Functioning : Preliminary Data in Healthy White Women. 2000;19(6):586-592.

3. Browne WJ, Lahi MG, Parker RM a. A Guide to Sample Size Calculations for Random Effect Models via Simulation and the MLPowSim Software Package School of Clinical Veterinary Sciences , University of Bristol * Tarbiat Modares University , Iran This draft – March 2009. 2009;(March).

4. Fritz MS, Mackinnon DP. Required Sample Size to Detect the Mediated Effect. *Psychol Sci*. 2007;18(3):233-239. doi:10.1111/j.1467-9280.2007.01882.x.Required

5. Laxy M, Teuner C, Holle R, Kurz C. The association between BMI and health-related quality of life in the US population: Sex, age and ethnicity matters. *Int J Obes*. 2018;42(3):318-326. doi:10.1038/ijo.2017.252

6. Galobardes B, Shaw M, Lawlor DA, Lynch JW, Smith GD. Indicators of socioeconomic position (part 1). *J Epidemiol Community Health*. 2006;60(1):7-12. doi:10.1136/jech.2004.023531

7. Krieger N, Williams DR, Moss NE. Measuring Social Class in US Public Health Research: Concepts, Methodologies, and Guidelines. *Annu Rev Public Health*. 1997;18(1):341-378. doi:10.1146/annurev.publhealth.18.1.341

8. Raftery AE. Bayesian Model Selection in Social Research. In: *Sociological Methodology , Vol . 25*. Vol 25. American Sociological Association; 1995:111-163.

9. Van Der weele T, Vansteelandt S. Mediation analysis with multiple mediators. *Epidemiol Method*. 2014;2(1):95-115. doi:10.1515/em-2012-0010

10. Andrews JC, Netemeyer RG, Burton S. The nutrition elite: Do only the highest levels of caloric knowledge, obesity knowledge, and motivation matter in processing nutrition ad claims and disclosures? *J Public Policy Mark*. 2009;28(1):41-55. doi:10.1509/jppm.28.1.41

1. Caloric knowledge was also measured in Study 2^10^. Because the scale did not reach adequate internal consistency (4 items – Cronbach’s α = 0.36), mediation through caloric knowledge was not examined. [↑](#footnote-ref-1)
